# An Incompletely Penetrant Novel Mutation in *COL7A1* Causes Epidermolysis Bullosa Pruriginosa and Dominant Dystrophic Epidermolysis Bullosa Phenotypes in an Extended Kindred

**DOI:** 10.1111/j.1525-1470.2012.01757.x

**Published:** 2012-04-20

**Authors:** Catherine S Yang, Yin Lu, Anita Farhi, Carol Nelson-Williams, Michael Kashgarian, Earl J Glusac, Richard P Lifton, Richard J Antaya, Keith A Choate

**Affiliations:** * Department of Dermatology; † Department of Genetics; ‡ Department of Pathology; § Howard Hughes Medical Institute, School of Medicine, Yale UniversityNew Haven, Connecticut

## Abstract

Epidermolysis bullosa pruriginosa (EBP) is a rare subtype of dystrophic epidermolysis bullosa (DEB) characterized by intense pruritus, nodular or lichenoid lesions, and violaceous linear scarring, most prominently on the extensor extremities. Remarkably, identical mutations in *COL7A1,* which encodes an anchoring fibril protein present at the dermal–epidermal junction, can cause both DEB and EBP with either autosomal dominant or recessive inheritance. We present one family with both dystrophic and pruriginosa phenotypes of epidermolysis bullosa. The proband is a 19-year-old Caucasian woman who initially presented in childhood with lichenoid papules affecting her extensor limbs and intense pruritus consistent with EBP. Her maternal grandmother saw a dermatologist for similar skin lesions that developed without any known triggers at age 47 and mostly resolved spontaneously after approximately 10 years. The proband’s younger brother developed a small crop of pruritic papules on his elbows, dorsal hands, knees, and ankles at age 13. Her second cousin once removed, however, reported a mild blistering disease without pruritus consistent with DEB. Genetic sequencing of the kindred revealed a single dominant novel intron 47 splice site donor G>A mutation, c.4668 + 1 G>A, which we predict leads to exon skipping. Incomplete penetrance is confirmed in her clinically unaffected mother, who carries the same dominant mutation. The wide diversity of clinical phenotypes with one underlying genotype demonstrates that *COL7A1* mutations are incompletely penetrant and strongly suggests that other genetic and environmental factors influence clinical presentation.

Dystrophic epidermolysis bullosa (DEB) is a clinically heterogenous class of diseases characterized by the formation of trauma-induced blistering at the sub-lamina densa level. Epidermolysis bullosa pruriginosa (EBP), first described by McGrath and colleagues in 1994 (1), is a rare subtype of DEB distinguished by intense pruritus, lichenified or nodular prurigo-like lesions, and violaceous linear scarring, most notably on the extensor extremities. Excoriations, milia, nail dystrophy, and albopapuloid lesions are often seen. Teeth and mucous membranes are usually unaffected. Although some cases of EBP are diagnosed within the first few years of life, many cases do not present until adulthood (2–7). As such, EBP can be mistaken for acquired disorders such as prurigo nodularis, lichen simplex chronicus, lichen planus, hypertrophic scarring, and dermatitis artefacta.

Dominant and recessive mutations in the gene *COL7A1* encoding for collagen VII have been reported to cause DEB. Collagen VII is the major anchoring fibril located below the basal lamina, at the dermal–epidermal junction. Dominantly inherited DEB typically presents with milder clinical symptoms and shows normal quantity and appearance of anchoring fibrils on biopsy. Recessive DEB is often more severe and usually results from premature termination codon mutations leading to decreased amounts of collagen VII apparent on electron microscopy and immunofluorescence staining.

The cause of pruritus in EBP is unknown. Attempts to correlate genotype with phenotype have been unsuccessful (7,10), since identical mutations in *COL7A1* can cause DEB in one patient and EBP in another (3,4,6,7,11–13). Alternate hypotheses regarding the origin of pruritus have included high serum immunoglobulin (Ig)E levels (3,24), concurrent filaggrin mutations leading to atopy (7), high expression of matrix metalloproteinase 1 (*MMP1*) causing imbalance of collagen degradation (16,18), and high cytokine interleukin (IL)-31 levels (10). None of these hypotheses uniformly account for pruritus observed in EBP.

We studied one kindred with both EBP and DEB phenotypes (Fig. 1). The proband is a 19-year-old woman of Italian descent who was referred to our clinic at age 5 with new-onset pruritic lesions on her right medial malleolus and chin. Over time, her pruritus gradually worsened, with extension of lesions to her extensor extremities bilaterally, the periumbilicus, the sacrum, and the upper back. No triggers were reported. She was otherwise healthy, without a history of atopy. Her parents are non-consanguineous, and neither reported any history of atopy, skin fragility, or pruritic dermatoses. The proband’s grandmother developed similar lesions at age 47 that persisted for approximately 10 years before mostly resolving spontaneously. The proband’s 15-year-old brother also developed pruritic papules at age 13 but was much less affected. Meanwhile, her second cousins once removed and their father reported only trauma-induced blistering. The objective of this study was to better characterize the genetic complexity of *COL7A1* mutations causing DEB and EBP in this kindred.

**Figure 1:**
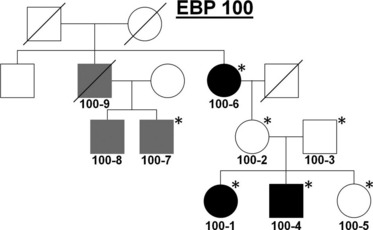
DEB and EBP phenotypes independently segregate in one kindred with incomplete penetrance. Individuals in EBP kindred 100 show either an epidermolysis bullosa pruriginosa phenotype (black symbols) or a dominant dystrophic epidermolysis bullosa phenotype (gray symbols). *Indicates genetic analysis has been performed.

## Methods

The Human Investigation Committee at Yale University approved the study protocol. Dermatologists clinically examined the proband, her immediate family members, and two members of her extended family (100–6, 100–7) between 1997 and 2011. In the proband, clinical diagnosis was verified with skin biopsies of lesional sites. IgE levels, thyroid function, renal function, liver function, iron, and ferritin were tested in the proband to exclude alternative causes of pruritus. Two family members reported to have DEB were unable to participate in this study (100–8, 100–9).

Genomic DNA was extracted from peripheral blood lymphocytes in the proband and immediate family members using phenol-chloroform extraction. Primer sets representing all 118 exons of *COL7A1*, including intron–exon boundaries, were generated with Primer3 (http://frodo.wi.mit.edu/primer3/) using the hg18 version of the genome masked to exclude DNA sequence repeats (GenomeMasker: http://bioinfo.ebc.ee/snpmasker/) as the template. We also generated primers for the five most common filaggrin mutations in European populations (R501X, 2282del4, 3702delG, R2447X, and S3247X) (17) and for the previously reported promoter single-nucleotide polymorphism (SNP) in *MMP1* [rs1799750: (−)>G] hypothesized to account for the pruritus of EBP (18). Polymerase chain reaction was conducted on 50 ng of DNA using KAPA2G Fast Polymerase (Kapa Biosystems, Woburn, MA) and its products were sequenced. Resulting sequences were compared with the human reference sequence (hg18, NCBI) using Sequencher (Gene Codes, Ann Arbor, MI). A single sequence variant in *COL7A1* was identified that was not found in SNP databases or in 95 ethnically matched unrelated controls without a history of skin disease.

Review of the literature for Table S1 was performed using the PUBMED search term “epidermolysis bullosa pruriginosa” between June 2009 and October 2011. Forty-two articles were identified; 13 were excluded because the case presented did not have EBP or because no genetic analysis was performed. Additional mutations were found using the Dystrophic EB Patient Registry (http://www.deb-central.org). Each mutation was then surveyed using the Human Gene Mutation Database (http://www.hgmd.org/) and recent reviews of the *COL7A1* gene (8,28) to determine whether it had been previously reported to cause DEB. Any discrepancies between sources were resolved by referring to the original article reporting the mutation in question.

## Results

**Figure 2:**
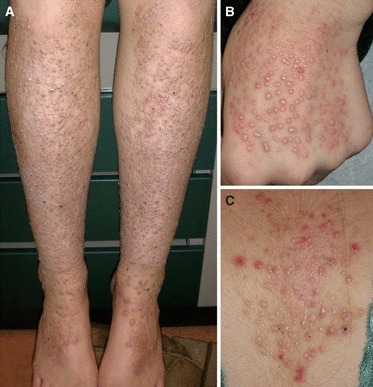
Clinical features of EBP in proband, 100-1. (A-C) Lichenoid papules and nodular prurigo-like lesions on shins bilaterally (A), dorsal hand (B), and upper back (C). Many papules are excoriated.

**Figure 3:**
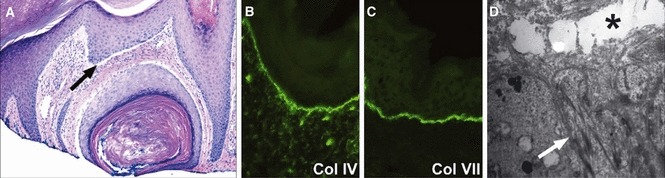
Histologic features of EBP in proband, 100-1. EBP is characterized by subepidermal clefting and disruption of anchoring fibrils. (A) Hematoxylin and eosin stain of affected skin shows subepidermal clefting (black arrow) and milium, 20X magnification. (B, C) Direct immunofluorescence staining shows normal pattern and amount of collagen IV and VII. (D) Electron microscopy of skin adjacent to a nodular lesion shows lysis and separation of collagen fibrils in the reticular dermis (white arrow). *Indicates subepidermal cleft.

On full skin examination, the proband displayed excoriated, lichenoid papules with milia coalescing into confluent plaques (Fig. 2). There was no nail dystrophy, and ancillary laboratory tests to evaluate her pruritus were all within normal limits. Skin tests for common food allergens and standard inhalants were negative except for an allergic reaction to penicillin at age 9. Histopathology of her lesions showed a sub-epidermal cleft with milia. Electron microscopy showed a sub-epidermal cleft with lysis and separation of collagen fibrils in the reticular dermis. There was no evidence of amyloid deposition. Direct immunofluorescence showed normal quantities and immunolocalization patterns of collagen IV, collagen VII, and keratin 14. Taken together, these findings supported a diagnosis of EBP (Fig. 3).

**Figure 4:**
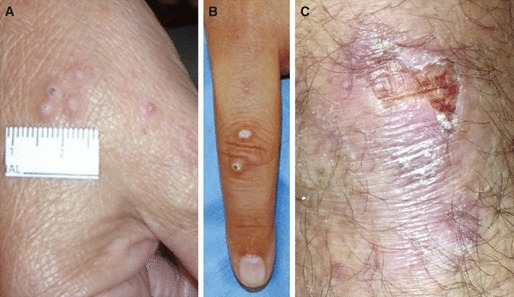
Clinical heterogeneity of EBP and DEB in kindred EBP 100. (A) Cluster of papules on the right hand of the proband’s maternal grandmother, 100-6. (B) Lichenoid papules on the left index finger of the proband’s 15-year-old brother, 100-4. (C) Blister and atrophic scarring on knee of the proband’s second cousin once removed, 100-7.

The only remnants of EBP in the propositus’ grandmother were macular scars on her upper back and intermittent clusters of pruritic papules on her upper extremities (Fig. 4A). The proband’s brother had few, isolated pruritic papules on his dorsal hands, elbows, knees, and ankles (Fig. 4B). The proband’s second cousin once removed had trauma-induced blistering, atrophic scarring, and few milia on his extensors (Fig. 4C).

**Figure 5:**
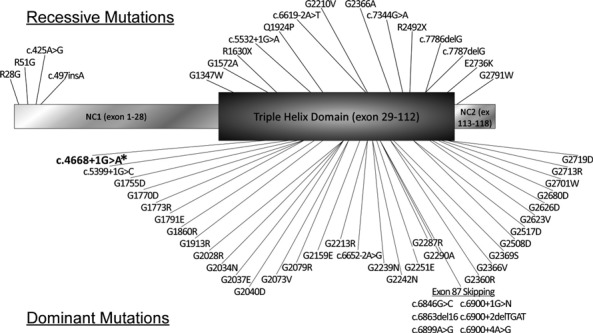
*COL7A1* mutations reported to cause EBP. Fifty-seven mutations are represented. Missense and nonsense mutations are represented using the protein sequence whereas splicing mutations, deletions, and insertions are represented using the cDNA sequence. The mutation identified in this family is bolded and denoted by an asterisk. Recessive mutations appear above the protein schematic and dominant mutations appear below.

Sanger sequencing of *COL7A1* in this kindred revealed a novel heterozygous splice site mutation in intron 47, c.4668 + 1 G>A, in the proband, her affected family members, and her unaffected mother. This mutation abolishes the intron 47 splice donor; similar mutations frequently lead to exon skipping or use of cryptic splice sites nearby (3,7,25,27,28). Sixty-two mutations causing EBP are reviewed in Fig. 5 and Table S1. All of the dominant mutations, including c.4668 + 1 G>A, interfere with the consecutive Gly-X-Y repeats in the triple helical domain of the protein. Ten recessive cases were identified and are summarized in Table S2.

Recognizing that filaggrin mutations could modify the phenotypes observed in this kindred (17), we performed sequencing of the five most common *FLG* variants in the European population in our patient and found none. We also addressed the findings of another group who suggested that a promoter SNP in *MMP1* leads to overexpression thus causing an imbalance of collagen degradation, with ensuing inflammation and pruritus, but sequencing of the proposed transcription factor binding site [rs1799750: (−)>G] of *MMP1* did not reveal any sequence differences between the proband and her mother.

## Discussion

No clear pattern emerges from attempts to correlate *COL7A1* gene mutations with disease phenotype (6,10,26,29). Most of the mutations in EBP are dominant, and many of the reported recessive cases may include a silent mutation or SNP in addition to a presumably causative coding mutation (Table S2). Incomplete penetrance is well documented in reports of unaffected family members carrying the same mutations as their affected relatives (4,7,13,22,23,31,32). For EBP, this phenomenon has been attributed to a presumed later onset, with the latest reported age being 71 (5). Although the proband and her brother presented in childhood, their grandmother developed EBP at age 47, and their 46-year-old mother currently has no skin findings despite carrying the same mutation.

The link between EBP and atopy is controversial. A history of atopy, in the probands themselves or in their relatives, might predispose some individuals to develop EBP instead of DEB, but genotypes associated with atopy such as filaggrin mutations and IL-31 polymorphisms are not consistently different between people with EBP and those with DEB (7,9,10). Some people with EBP have high IgE levels, but most have normal levels (3).

Affected individuals can transition from a DEB to an EBP phenotype; most attribute this to the development of pruritus, which in turn causes the lesions of EBP (3,7,13–15). This finding lends support to reports that question whether EBP is a unique disease entity or whether the phenotype is due to pruritus superimposed on DEB (1,5,31). Several cases in the literature suggest an initiating event for pruritus such as atopic dermatitis or allergies, thyroid dysfunction, and even varicella zoster virus infection (3,7). However, in most instances, no cause is found. Neither the index case nor her brother has atopy, and although their grandmother developed hypothyroidism in her 30s, she was clinically euthyroid at the time of her EBP eruption nearly 15 years later.

**Figure 6:**
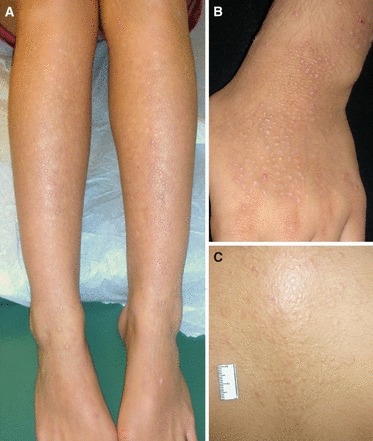
Improvement of skin lesions with systemic thalidomide administration. The index case was started on thalidomide 100 mg daily and improved gradually with most apparent results at two months. Shins (A), left dorsal hand (B), and mid-upper back (C) show flattening of papules with less excoriation after 6 months of treatment.

EBP is notoriously challenging to treat (19,20,30). Our patient was treated with numerous medications, including topical and systemic corticosteroids, antihistamines, anti-opiates, tumor necrosis factor alpha (TNF-α) inhibitors, and phototherapy with minimal success. Oral cyclosporine 5 mg/kg mildly improved her pruritus but was discontinued after 5 months because of marked gingival hyperplasia. Thalidomide has been documented to improve EBP, most likely through immunomodulation of the production of TNF-α, interferon gamma, and other cytokines (11,21). Our patient was started on 100 mg/day of thalidomide and reported decreased pruritus and flattening of her lesions within 2 months. She developed mild peripheral neuropathy after 4 months of treatment, so the dose was decreased to 50 mg/day with resolution of her paresthesia and continued efficacy. After 9 months of thalidomide treatment, the proband’s pruritus is well controlled, and her lesions continue to flatten, leaving hypopigmented macules and milia (Fig. 6).

This case demonstrates the phenotypic complexity and incomplete penetrance of *COL7A1* mutations in an extended kindred with DEB and EBP. The same mutation in intron 47, c.4668 + 1 G>A, resulted in mild EBP in one individual, a severe but time-limited course in another, and no effect in yet another, but caused severe EBP in the proband that responded to thalidomide treatment after more than 10 years of trying alternative therapies. Additionally, the same mutation caused classic DEB with solely trauma-induced blistering in another branch of this family. Clinical studies and DNA sequencing for five common European variants of filaggrin and an *MMP1* promoter SNP failed to reveal the etiology of the proband’s pruritus. Given that the observed mutation is predicted to affect splicing, one might speculate that alternative splice isoforms are expressed in different members of this kindred, though this would not explain the phenotypic variability seen in the majority of EBP kindreds carrying missense mutations. We expect that genetic and/or environmental factors are involved in the initiation and progression of EBP, although the exact mechanisms remain to be determined. Further elucidation may become permissible with broader availability of whole genome and transcriptome sequencing, and better understanding of common polymorphisms within the population that may contribute to the pathogenesis of pruritus and to EBP.

## Supporting Information

**Table S1** Reported EBP mutations organized by position within *COL7A1*. **Table S2** Reported mutations causing recessive EBP

Please note: Wiley–Blackwell are not responsible for thecontent or functionality of any supporting materials supplied bythe authors. Any queries (other than missing material) should bedirected to the corresponding author for the article.
