# Supplementary material for: An Incompletely Penetrant Novel Mutation in COL7A1 Causes Epidermolysis Bullosa Pruriginosa and Dominant Dystrophic Epidermolysis Bullosa Phenotypes in an Extended Kindred
Source: Pediatr Dermatol. 2012 Apr 20;29(6):725–31. doi: 10.1111/j.1525-1470.2012.01757.x (PMC3709244; doi:10.1111/j.1525-1470.2012.01757.x)
Supplement: Supplementary file 1 — Table S1 Reported EBP mutations organized by position within COL7A1. Table S2 Reported mutations causing recessive EBP [file pde0029-0725-sd1.pdf]

Mutation	Exon	Effect	Reported to cause DEB	Age of onset	Ethnicity	Sex	IgE level	Other affected individuals	Reference
p.R28G/ p.G2366A	1/92	Missense/ Missense	G2366N: RDEB-nHS	DEB since 8 yo; EBP developed after first pregnancy	Italian	F	Normal	Brother with nail-dystrophy-only DEB	Pruneddu, 2010
p.R51G/ p.R2492X	2/98	Missense/ PTC	c.7474C>T: RDEB-nHS	13 yo	Italian	M	Normal	None	Drera, 2006
c.425A>G/ c.7344G>A	3/95	Splice PTC/ Splice PTC	Both: RDEB-nHS	12 yo	Italian	F	Normal	None	Drera, 2006
c.425A>G/ p.E2736K	3/110	Splice PTC/ Missense	c.425A>G: RDEB-nHS	DEB since 6 yo; EBP developed at 53 yo	Caucasian	M	Normal	None	Schumann, 2008
c.497insA/ p.G1347W	4/34	PTC/ Missense	c.497insA: RDEB-HS G1347N: RDEB-nHS	8 yo	Caucasian	F	Normal	None	Schumann, 2008
c.4668+1 G>A	47	In-frame exon skipping	No	5 yo	Italian	F	Normal	Multiple family members w/ DDEB spectrum	This report
p.G1572A/ c.7787delG	48/104	Missense/ PTC	No	NA	NA	NA	NA	NA	Almaani, 2011
p.R1630X/ c.7344G>A	51/95	Nonsense/ Splice PTC	R1630X: RDEB-HS c.7344G>A: RDEB-nHS	8 yo	Italian	F	Normal	None	Drera, 2006
p.R1630X	51	Nonsense	RDEB-HS	1 yo	Caucasian	F	Normal	None	Schumann, 2008
c.5399+1G>C	In. 55	In-frame exon skipping	No	6 yo	Caucasian	M	NA	Mother and sister w/ toenail dystrophy	Tey, 2011
p.G1755D	59	Missense	No	NA	NA	NA	NA	NA	Posteraro, 2005
p.G1755D	59	Missense	No	39 yo	Italian	F	Normal	None	Drera, 2006
p.G1755D	59	Missense	No	8 yo	Turkish	F	Normal	Mother with nail-dystrophy-only DEB	Schumann, 2008
p.G1770D	61	Missense	RDEB	NA	NA	NA	NA	NA	Almaani, 2009
p.G1773R	61	Missense	DDEB	9 yo	Chinese	M	Normal	Mom and maternal grandfather w/ mild phenotype	Jiang, 2011
p.G1791E	61	Missense	No	Infancy	Caucasian	F	NA	None	Mellerio, 1999
c.5532+1G>A/ c.7786delG	In 64/ Ex104	In-frame exon skipping/ PTC	c.7786delG: RDEB-nHS	Infancy	Caucasian	F	NA	None	Mellerio, 1999
p.G1860R	66	Missense	No	NA	NA	NA	NA	NA	Almaani, 2009

p.G1913R	69	Missense	No	NA	NA	NA	NA	NA	Almaani, 2009
p.Q1924P/ c.6619-2A>T	Ex 69/ In 82	Missense/ In-frame exon skipping	No	NA	Middle Eastern	NA	NA	NA	Abu Sa'd, 2006
p.G2028R	73	Missense	DDEB and RDEB	20 yo	Japanese	F	NA	Father, aunt, and paternal grandmother with EBP	Murata, 2000
p.G2028R	73	Missense	DDEB and RDEB	DEB since 1 yo; EBP developed at 18 yo	Caucasian	F	Normal	Grandmother, brother, and daughter with DEB	Schumann, 2008
p.G2034R	73	Missense	DDEB	NA	Chinese	NA	NA	NA	Chen, 2000
p.G2034W	73	Missense	DDEB	DEB since birth; EBP developed at 7 yo	Caucasian	F	Normal	Grandmother, father, and sister have DEB	Schumann, 2008
p.G2037E	73	Missense	DDEB	NA	NA	NA	NA	NA	Unpublished
p.G2040D	73	Missense	DDEB	Infancy	Caucasian	F	Elevated	None	Ozanic, 2005
p.G2073V	75	Missense	RDEB-nHS	10 yo	Italian	F	Elevated	Father with EBP	Drera, 2006
p.G2079R	75	Missense	No	NA	NA	NA	NA	NA	Abu Sa'd, 2006
p.G2159E	79	Missense	No	NA	NA	NA	NA	NA	Almaani, 2009
p.G2210V/ p.G2791W	83/113	Missense/ Missense	G2791W: DDEB	NA	Aboriginal	NA	NA	Multiple family members w/ DDEB-Pr and Pasini subtype	Dang, 2007
p.G2213R	83	Missense	RDEB	37 yo	Caucasian	F	NA	NA	Almaani, 2009
c.6652-2A>G	83	In-frame exon skipping	No	NA	NA	NA	NA	NA	Abu Sa'd, 2006
p.G2239D	85	Missense	No	NA	NA	NA	NA	NA	Tamai, 1998
p.G2239V	85	Missense	No	NA	NA	NA	NA	NA	Almaani, 2011
p.G2242E	85	Missense	No	NA	NA	NA	NA	NA	Tamai, 1998
p.G2242E	85	Missense	No	12 yo	Japanese	F	NA	Son w/ bullous dermolysis of the newborn	Murase, 2011
p.G2242R	85	Missense	No	Teens	Chinese	F	NA	Father, brother, aunt, and cousin affected with mild EBP	Lee, 1997
p.G2242R	85	Missense	No	7 yo	Caucasian	M	NA	Father with mild EBP	Mellerio, 1999
p.G2242W	85	Missense	No	10 yo	Chinese	M	Elevated	Father, grandfather, and paternal uncle with EBP	Shi, 2009

p.G2251E	86	Missense	DDEB	25 yo	Chinese	F	Normal	4 siblings and mother with EBP; 2 unaffected siblings with mutation	Ee, 2007
p.G2251E	86	Missense	DDEB	1 month	Japanese	M	NA	Unaffected father w/ mutation	Takiyoshi, 2011
c.6846G>C	87	In-frame exon skipping	No	3 kindreds	Danish	NA	NA	Multiple family members w/ blistering and nail dystrophy	Covaciu, 2011
p.G2287R	87	Missense	DDEB	71 yo	Japanese	M	Normal	None	Hayashi, 2011
c.6863del16	87	PTC	No	11 yo	Hispanic	M	NA	Daughter with mild EBP; 5 generation family with DEB	Mellerio, 1999
p.G2290A	87	Missense	No	40s	Caucasian	F	NA	NA	Almaani, 2009
c.6899A>G	87	In-frame exon skipping	No	< 35 yo	Chinese	M	NA	18 other family members with EBP	Jiang, 2002
c.6900+1G>T	In. 87	In-frame exon skipping	DEB	2 months	Chinese	M	Normal	11 other family members with EBP	Ren, 2007
c.6900+1G>C	In. 87	In-frame exon skipping	No	20 yo	Chinese	M	Normal	Multiple affected family members	Jiang, 2011
c.6900+2delTGAT	In. 87	In-frame exon skipping	No	30 yo	Italian	F	Elevated	16 members in 5 generations with skin lesions	Drera, 2006
c.6900+4A>G	In. 87	In-frame exon skipping	RDEB-HS	38 yo	Italian	F	Normal	5 other affected members in 4 generations with skin lesions	Drera, 2006
p.G2360R	92	Missense	No	NA	NA	NA	NA	NA	Almaani, 2011
p.G2366V	92	Missense	RDEB-nHS	20s	Chinese	F	Normal in proband; elevated in son	Son and daughter with EBP	Chuang, 2004
p.G2369S	93	Missense	RDEB	17 yo	Pakistani	M	NA	None	Mellerio, 1999
p.G2508D	100	Missense	No	NA	NA	NA	NA	NA	Almaani, 2011
p.G2517D	100	Missense	No	NA	NA	NA	NA	NA	Almaani, 2011
p.G2623V	105	Missense	RDEB-nHS and DDEB	8 yo	Caucasian	M	Normal	Brother with DEB	Schumann, 2008
p.G2626D	106	Missense	No	2 yo	Chinese	F	NA	4-generation family with skin lesions	Wang, 2007
p.G2680D	108	Missense	No	NA	NA	NA	NA	NA	Almaani, 2011

p.G2701W	109	Missense		25 yo	Chinese	F	Normal	Unaffected mother w/ mutation	Jiang, 2011
p.G2713R	110	Missense	DDEB	29 yo	Caucasian	F	NA	Father and nephew with DEB	Mellerio, 1999
p.G2713R	110	Missense	DDEB	27 yo	Turkish	F	NA	None	Broekaert, 2006
p.G2719D	110	Missense	No	6 yo	Caucasian	F	NA	Brother and mother	Riedl, 2009

Supplementary Table 1. Reported EBP mutations organized by position within *COL7A1*. We found 62 reports of EBP. In 27 cases, other family members were affected with DEB and its variants. Of the 57 different mutations found, 34 mutations were not previously reported to cause either DDEB or RDEB. All probands have unifying features of pruritus, lichenoid papules, and/ or prurigo-like nodules most commonly on the shins. Many additionally presented with milia and nail dystrophy. Missense mutations are denoted using protein nomenclature while other mutations consisting of, but not limited to, splice site mutations, insertions, and deletions are denoted using the coding sequence. AR = autosomal recessive; AD = autosomal dominant; DDEB = dominant dystrophic epidermolysis bullosa; RDEB-HS = Hallopeau-Siemens recessive dystrophic epidermolysis bullosa; RDEB-nHS = non-Hallopeau-Siemens recessive dystrophic epidermolysis bullosa; In = intron; X = termination codon; N = any protein; YO = years old; NA = not available; ND = not detected.

Case	Mutation 1	Mutation 2
1	p.R28G (missense)	**p.G2366A** (missense)
2	**p.R51G** (missense)	p.R2492X (PTC)
3	c.425A>G (splice PTC)	**c.7344G>A** (splice PTC)
4	c.425A>G (splice PTC)	**p.E2736K** (missense)
5	c.497insA (PTC)	**p.G1347W** (missense)
6	**p.G1572A** (missense)	c.7787delG (PTC)
7	p.R1630X (nonsense)	**c.7344G>A** (splice PTC)
8	**c.5532+1G>A** (exon skipping)	c.7786delG (PTC)
9	p.Q1924P (missense)	c.6619-2A>T (in-frame exon skipping)
10	p.G2210V (missense)	**p.G2791W** (missense)

Supplementary Table 2. Reported mutations causing recessive EBP. The majority of these cases have one mutant allele predicted to result in loss of function and a second gain of function mutant allele expected to contribute to disease phenotype, giving essentially mono-allelic expression. Exceptions include cases 1, 9, and 10 in which two missense mutations are present. The mutations c.425 A>G, c.497insA, and R2492X are predicted to cause nonsense-mediated decay and thus would not be expressed. In Case 10, the authors postulated that previously reported G2791W was the relevant mutation and that G2210V is a polymorphism found in Australian Aboriginals (8). The putative causative mutations are bolded, based on prior reports of the same mutation causing EBP or presumed deleterious effect on the encoded protein. PTC=premature termination codon
